# *Programa de diabetes*: improving diabetes care for undocumented immigrants using the Chronic Care Model at a free community clinic

**DOI:** 10.1007/s00592-023-02084-1

**Published:** 2023-04-10

**Authors:** Mairi Gael Leining, Xiaobin Zhou, Gayane Yenokyan, Shaunicy Sturm, Jennifer Meyer, Yomira Diaz, MaeLin Sorenson, Nina Chartrand

**Affiliations:** 1 People’s Health Clinic, Johns Hopkins Bloomberg School of Public Health, Park City, Baltimore, UT, MD USA; 2 Johns Hopkins Bloomberg School of Public Health, Baltimore, USA; 3 People’s Health Clinic, Park City, USA

**Keywords:** Undocumented immigrants, Free community clinic, Diabetes management, Social determinants of health, Health equity

## Abstract

**Aims:**

This study examined whether the Chronic Care Model can be successfully applied to improve health outcome measures for uninsured, undocumented immigrants with diabetes at a free, non-federally funded community clinic.

**Methods:**

Data were collected from 128 uninsured, undocumented immigrants enrolled in *Programa de diabetes*, a comprehensive diabetes program at People’s Health Clinic based on the six core elements of the Chronic Care Model. All study participants self-identified by the Hispanic ethnicity. A longitudinal study design was used to compare baseline diabetic health measures with outcome data after patient program participation over a 12-month enrollment period. Linear mixed effect model was used to determine the patient specific change in HbA1C across time, controlling for gender, age, food insecurity, income level, diabetes type, and literacy. In addition, McNemar tests were conducted to compare the coverage of eye exams and statin use before and after program enrollment.

**Results:**

After program enrollment, individual specific change in HbA1C was expected to be − 0.201 [95% CI 0.244, − 0.158] % per month after controlling for baseline covariates. There were statistically significant improvements in both eye exam coverage (*p* < 0.01) and statin use (*p* < 0.01).

**Conclusions:**

The Chronic Care Model can be successfully applied to improve health outcome measures at a free, non-federally funded community clinic among uninsured, undocumented immigrants, who identify by the Hispanic ethnicity and have the diagnosis of diabetes. Barriers to care including food insecurity, federal poverty level and illiteracy do not preclude glycemic control.

## Introduction

An estimated 34.1 million adults have diabetes in the United States with an increasing trend in prevalence [1]. It is well established that diabetes disproportionately affects people of lower socioeconomic status and ethnic minorities are more likely to experience diagnostic and treatment delays [2, 3]. Adults who identify by the Hispanic ethnicity, the largest ethnic minority group in the United States, are 70% more likely to develop diabetes and experience the complications of diabetes than non-Hispanic white adults [1, 4].

Several studies have demonstrated the efficacy of multidisciplinary, community-based diabetes programs that specifically address the sociocultural needs of Hispanic patients. Influenced by the landmark study, Project Dulce, many of these programs utilize the Chronic Care Model including Diabetes Self-management Education and Support (DSMES) to achieve significant improvements in diabetic health outcomes [5–9]. However, studies of Hispanic adults with diabetes often include a diverse group of people with varying countries of origin, insurance coverage, and residency status. Many have suggested that further research should be done to evaluate diabetes management among subsets of patients identified within the broad categorization of the Hispanic ethnicity [3, 5].

In the United States, approximately 21% of the Hispanic population are undocumented immigrants, the majority of whom are from Mexico [10]. Undocumented immigrants are particularly vulnerable to the complications of diabetes due to several complex factors including poverty, food insecurity and legal barriers to quality health care. With undocumented legal status, they are not eligible for the Affordable Care Act or Medicaid. Many work more than one job yet live below the federal poverty level, which is only $13, 590 per year for a single adult. Their lack of residency status leads to exploitation in the workplace including unsafe working conditions and labor trafficking [11–13].

At People’s Health Clinic, approximately 80–85% of our patients are undocumented residents and 100% of our patients are uninsured. In 2019, People’s Health Clinic provided care to 193 adult patients with diabetes; 40% of these patients at intake had a HbA1C > 9.0%, placing them at high risk for the complications of uncontrolled hyperglycemia such as cardiovascular disease, chronic kidney disease and vision loss.

The objective of this study is to determine whether health outcome measures can be improved among undocumented immigrants with diabetes at a free, non-federally funded clinic by utilizing the Chronic Care Model framework to provide high-quality, patient-centered care [14–16].

## Research design and methods

### Study design

A longitudinal research design was used for this study. Baseline data were obtained at the time of patient enrollment in our diabetes program, *Programa de diabetes*, and followed-up to outcome data after 12 months of program inception. All data were collected between the dates of October 1, 2020, and October 1, 2021. Approval for this quality improvement project was obtained from the People’s Health Clinic Board of Directors.

### Study setting and population

People’s Health Clinic is a rural, non-profit, non-federally funded, private community clinic that serves the uninsured residents of Summit and Wasatch Counties, Utah. Most of our patients self-identify by the Hispanic ethnicity and are undocumented immigrants who are not eligible for government-sponsored health insurance programs. Inclusion criteria in *Programa de diabetes* was as follows: uninsured adult patients age >  = 18-years-old with a known diagnosis of diabetes. 193 patients at People’s Health Clinic were initially identified by Athena, the electronic medical record (EMR), as having a prior diagnosis of diabetes. Although still invited to receive comprehensive diabetes care through our diabetes program, patients were excluded from this study if they had legal residency status, pre-diabetes, did not identify by the Hispanic ethnicity, or were age < 18-years-old at time of enrollment.

### Measures

At time of program enrollment, patients’ medical records were reviewed, and the diagnosis of diabetes was verified by prior HbA1C data available through the EMR. Diabetes was defined by two prior HbA1C results >  = 6.5%. We determined patient demographic characteristics using the Chronicle Diabetes Assessment Form [17]. Patients also completed a validated food insecurity screen and provided the clinic with income verification used to determine their Federal Poverty Level [18, 19]. The clinical outcome measures evaluated in this study include HbA1C, statin use, weight, and diabetic retinopathy screening.

### Procedures

We applied the Chronic Care Model (CCM) framework to our diabetes program by implementing the six core elements of the CCM as listed below [14].

1. *Delivery system design (moving from a reactive to a proactive care delivery system where planned visits are coordinated through a team-based approach*)

We created the *Programa de diabetes* team led by a volunteer internal medicine physician and a nurse diabetes educator, who was contracted to work for 24 h per week. The team also included staff and volunteer medical providers, a case manager, a clinic coordinator, a medical assistant, and a volunteer pharmacist. Our annual budget was $225,000 US dollars total with $215,000 for staff expenses, and $10,000 for diabetes program supplies paid for through private community donations. Our goal as a team was to make diabetes care as accessible as possible for our patients.

On program enrollment, patients were seen by the diabetes educator in consultation with the internal medicine physician. Each new patient would complete the Chronicles Diabetes Assessment form, food insecurity screen and provide income verification with either a tax return or two prior income statements. Each patient received a *Programa de diabetes* folder, which included a welcome letter that explained the purpose of the program, American Diabetes Association (ADA) educational brochures, and a glucose log. We provided all patients with free diabetic supplies including glucometers, test strips, lancets, and insulin needles if applicable. Patients received their medication list on a form that we specifically designed for our program, and we color-coded medications if patients screened positive for illiteracy. Patients also received a card with the image of a HbA1C-meter, that places patients in a colored zone depending on HbA1C level. The card included current HbA1C, goal HbA1C range, and follow-up appointment information.

We scheduled follow-up appointments with the patients in the room so we could ensure that they were scheduled at a time that best fit their work and family schedules. Patients were scheduled to be seen by the diabetes educator in coordination with their primary care provider at least every 3 months. Patients who were considered high risk due to limited health literacy or more complicated medication regimens were seen as frequently as needed, sometimes weekly. Any patient who was prescribed insulin was seen for an additional visit within 1 week and was placed on a weekly phone call follow-up list. If a patient missed an appointment, we called them that day to reschedule to ensure that they were not lost to follow-up.

2. *Self-management support*

People’s Health Clinic hired a diabetes educator for *Programa de diabetes*. The medical director and diabetes educator enrolled in the ADA Education Recognition Program (ERP) in September 2020, which provided access to instructional webinars for DSMES program implementation. In December 2020, after completing one Initial Comprehensive DSMES Cycle ^(17)^, we received certification from the ADA as a recognized DSMES program. Using the ERP platform, Chronicles Diabetes, our diabetes educator recorded hours of DSMES provided to patients, which were all conducted as one-on-one visits. DSMES education was individualized to the patients’ health literacy levels, responses to the Chronicle Diabetes Assessment questionnaire, and complexity of medications.

3. *Decision support (basing care on evidence-based, effective care guidelines*)

Prior to implementing the diabetes program, the medical director reviewed the ADA “Standards of Medical Care in Diabetes—2020” to ensure best clinical practice. Based on the ADA guidelines, the medical director created clinic practice protocols for the other providers. At time of program enrollment, the patient’s laboratory studies were updated, and vaccination status was addressed. Medication management was customized to each patient by a combination of laboratory results, patient preferences, and feasibility of medication access. When necessary, patients completed Patient Assistance Program (PAP) applications during their appointments to qualify for free medications including some types of insulin, GLP1-agonists, SGLT2-inhibitors, and DPP4-inhibitors. Our patients’ access to free medications from pharmaceutical companies was restricted to companies that did not require legal status for application submission. For example, we were able to obtain PAP medications from Sanofi, Merck, and Boehringer Ingelheim, but not Eli Lilly or Novo Nordisk. Additionally, patients purchased low-cost, generic medications through local pharmacies or received medications donated by Direct Relief, a program that reroutes excess medication inventory to free clinics. All patients age >  = 40-years-old were prescribed a statin unless contraindicated. Patients were referred to ophthalmology for annual diabetic eye exams.

4. *Clinical information systems (using registries that can provide patient-specific and population-based support to the care team)*

We used the Athena EMR to identify our diabetic patients by diagnosis of type 1 or type 2 diabetes and HbA1C results prior to program enrollment. Athena was also used for provider documentation and all orders including medications, vaccinations, and laboratory studies. The ADA platform, Chronicle Diabetes, was used by our diabetes educator to record DSMES visits, and hours of DSMES instruction received by patients. All patients enrolled in *Programa de diabetes* were added to Chronicle Diabetes, so it could serve as our comprehensive list of program participants.

5. *Community resources and policies (identifying or developing resources to support healthy lifestyles)*

To expand patient services, we relied on established partnerships and developed new relationships with local non-profit community organizations. These partnerships were especially critical for us to address the potential barriers to diabetes care identified by the Chronicles Diabetes Assessment Form. For example, patients who screened positive for food insecurity were referred to the Christian Center of Park City, which runs a food bank. Patients who required Medicaid denial letters to qualify for PAP programs, received application support from community health workers at Holy Cross Ministries.

6. *Health systems (to create a quality-oriented culture)*

People’s Health Clinic depends on partnerships with local health systems including Intermountain Healthcare and University of Utah. Intermountain Healthcare provides substantial financial support to People’s Health Clinic by covering the cost of all laboratory studies. Intermountain Healthcare also provides free consultations with subspecialty providers including endocrinology, neurology, and cardiology. The Moran Eye Center at the University of Utah provides a monthly evening clinic for diabetic eye exams.

### Statistical analysis

The analysis included 128 uninsured, undocumented immigrant patients. The primary analytic objective was to assess the change in HbA1C during one year of the program participation. The relationship between HbA1C and time since program enrollment was explored overall and separately by income level and food insecurity status. Linear mixed effect model was fit to the data to determine the patient-specific change in HbA1C after enrolling in the program, controlling for gender, age, food insecurity, income level, diabetes type, and literacy. Potential effect modifications between program enrollment and above covariates were explored stepwise. ANOVA test was used to determine whether to add the covariates as effect modifiers in the final model. Similar (secondary) analysis was conducted for weight and BMI change. Additionally, McNemar tests were conducted to compare whether the coverage of eye exams and statin use for the participants was different before and after enrolling into the program while accounting for the paired nature of the data; and to compare types of diabetes medications used by the participants before and after program enrollment. The analyses were conducted using R 4.0.4.

## Results

Summary of characteristics of participants at baseline is shown in Table 1. The majority of 128 participants included in the analysis were greater than 45 years of age (62.5%) and 84 (66%) of them were female. 74 (58%) of the participants screened positive for at risk of food insecurity and 45 (35%) participants were more than 100% above federal poverty level. 125 out of 128 (98%) participants were diagnosed with Type II diabetes. At the time of study enrollment, the median HbA1C was 9.05 [IQR 7.30, 10.90] %. Among 117 participants with known weight, the median weight was 73.03 [IQR 62.14, 84.82] kg.

**Table 1:** Characteristics of patients at baseline

Characteristic	*N* = 128*
*Gender*	
Female	84 (66%)
Male	44 (34%)
Age	
18–45	48 (38%)
46–65	75 (59%)
> 65	5 (3.9%)
Food insecure	74 (58%)
*Income level in percentage of federal poverty level*	
< 100%	83 (65%)
101–200%	41 (32%)
> 200%	4 (3.1%)
Type 2 Diabetes	125 (98%)
Illiterate	25 (20%)
HbA1C (%)	9.05 (7.30, 10.90)
Weight/kg†	73.03 (62.14, 84.82)

*n (%); Median (IQR)

†*N* = 117

As shown in Table 2, for a specific individual, the HbA1C was expected to decrease statistically significantly by 0.201% for each additional month in the study [− 0.201, 95% CI − 0.244, − 0.158]. The intraclass correlation coefficient was 0.406, meaning that 40.6% of the variance in the measurements of HbA1C comes from the variability among individuals. After controlling for gender, age, food insecurity, income level, diabetes type, illiteracy, for a specific individual, the HbA1C was expected to decrease statistically significantly by 0.203 [− 0.203, 95% CI − 0.246, − 0.160] % per month. No statistically significant differences in HbA1C at baseline were found by gender, age, food insecurity, income level, diabetes type, and literacy status. In our analyses, these baseline covariates did not modify the relationship between duration of follow-up and change in HbA1C either. We did not find statistically significant changes in weight and BMI of participants since they enrolled in the program.

**Table 2:** Individual specific effect of program and other baseline covariates on HbA1C (%)

	Estimate	SE	95% Confidence Interval		*p* value
*Model 1: Not adjusted for baseline covariates*					
Baseline	9.090	0.160	8.774	9.406	< 0.001
Duration in Program/Month	− 0.201	0.022	− 0.244	− 0.158	< 0.001
*Model 2: Adjusted for baseline covariates*					
Baseline	10.394	0.978	8.457	12.332	< 0.001
Duration in Program/Month	− 0.203	0.022	− 0.246	− 0.160	< 0.001
*Gender*					
*Male*	0.188	0.314	− 0.434	0.811	
Age					
*46–65*	0.002	0.312	− 0.616	0.619	
> *65*	− 0.634	0.795	− 2.209	0.940	
Food insecure	− 0.205	0.296	− 0.792	0.381	
*Income level in percentage of federal poverty level*					
*101–200%*	− 0.404	0.311	− 1.021	0.213	
> *200%*	1.329	0.833	− 0.322	2.980	
Type II Diabetes	− 1.282	0.964	− 3.192	0.628	
Illiterate	0.604	0.378	− 0.144	1.352	

Table 3 shows the results of comparison of eye exams and statin use for individuals before and after enrollment in the program. There was statistically significant improvement in eye exam coverage for participants after they enrolled into the program (*p* < 0.01). In addition, the program also improved statin use statistically significantly; 25.8% of the participants did not use statin before enrolling in the program but used statin after enrolling into the program (*p* < 0.01).

**Table 3:** Comparison of received care before and after program enrollment

		After		*p* value*
		No	Yes	< 0.01
*Eye exam*				
Before	No	31 (24.2%)	78 (60.9%)	
	Yes	8 (6.3%)	11 (8.6%)	
*Use of statin*				
Before	No	20 (15.6%)	33 (25.8%)	
	Yes	0 (0%)	75 (58.6%)	

*p value is calculated from McNemar Test

Table 4 shows a comparison of types of medications prescribed before and after program enrollment. There was a statistically significant increase in the number of patients who received a biguanide, SGLT2 inhibitor, GLP-1 agonist, DPP-4 inhibitor, or long-acting insulin after program enrollment (*p* < 0.01). 84% of patients received free medications through the completion of PAP applications during this study.

**Table 4:** Comparison of medication types administered before and after program enrollment

Medication type	Before	After	*p* value*
Biguanide	99 (77%)	119 (93%)	< 0.001
SGLT2 Inhibitor	17 (13%)	61 (48%)	< 0.001
GLP-1 Agonist	6 (5%)	30 (23%)	< 0.001
DPP-4 Inhibitor	30 (23%)	54 (42%)	< 0.001
Sulfonylurea	7 (5%)	13 (10%)	0.15
Insulin (long-acting)	38 (30%)	58 (45%)	< 0.001
Insulin (short-acting)	4 (3%)	5 (4%)	1.00

*p value is calculated from McNemar Test

## Conclusions

Implementing a comprehensive diabetes program using the Chronic Care Model framework was effective in improving health outcomes among undocumented immigrants with diabetes at a free, non-federally funded community clinic. These results demonstrate a significant improvement in HbA1C despite barriers to care such as food insecurity, federal poverty level or illiteracy. A significant reduction in BMI was not detected. A potential explanation for this may be that several patients began insulin therapy during the program, which is associated with weight gain. These findings are consistent with prior health outcome measures reported by Project Dulce and Project Dulce-associated clinical trials [7–9]. In addition, the results show a significant improvement in the proportion of patients who received annual diabetic eye exams.

This study is unique because we only include uninsured, undocumented immigrants who identify as Hispanic in the data analysis, whereas prior studies have included patients who identify by the Hispanic ethnicity and have health insurance due to their legal residency status in the United States. Undocumented immigrants struggle with more extreme barriers to quality healthcare access, especially due to their ineligibility for government healthcare programs, and restricted access to private pharmaceutical company assistance programs. As a result, undocumented immigrants are less likely to receive the standard of care for the prevention and management of chronic conditions like diabetes than legal residents [3–5]. Furthermore, undocumented immigrants are at increased risk for poverty and food insecurity, compounded by their exclusion from state and federal unemployment, childcare, and food assistance programs. Undocumented immigrants are also more likely to struggle with language barriers, low-educational status, housing insecurity and access to reliable transportation. Therefore, residency status itself is a social determinant of health [12].

Despite the additional barriers to care faced by undocumented immigrants, we showed a statistically significant improvement in HbA1C, even when HbA1C results were adjusted for food insecurity, federal poverty level and illiteracy. These results suggest that the social determinants of health (including residency status, poverty level and food insecurity) do not preclude quality diabetes management. The chronic care model can be successfully applied to this patient population with demonstrable outcomes that may reduce the complications of diabetes. However, we found that we must remain flexible in how we adjust the chronic care framework to meet the unique needs of our patient population. The People’s Health Clinic patients responded positively to our model that provided 1:1, in-person care, with a combination of physician-guided, customized medical management and nurse educator-supported DSMES during each appointment.

Limitations of our diabetes program included restricted access to best-practice medications such as preferred GLP-1 agonists since some pharmaceutical companies require that patients have legal residency status to apply for patient assistance programs. For example, the only GLP-1 agonist available to our patients through patient assistance programs was lixisenatide. As a result, we had to adjust medication regimens in favor of price and availability over efficacy and patient risk factor profile. In addition, because we are a non-federally funded program dependent on private donations, we have not yet secured the funding for community health workers as used in the Project Dulce model [9]. With additional funding and expanded access to medications, People’s Health Clinic believes that we could achieve an even greater improvement in diabetic health outcome measures in our target population.

Ultimately, undocumented residents with diabetes would benefit from expanded government subsidies to ensure quality health care access. In this current healthcare environment, we must provide comprehensive, patient-centered care to the most vulnerable members of our communities if we hope to impede the diabetes epidemic.
